# Effect of wavelength and radiant emittance on light transmittance and temperature increase in dental resin-based composites

**DOI:** 10.1590/1678-7757-2025-0307

**Published:** 2025-10-08

**Authors:** Sharanya Singh, Mateus Garcia Rocha, Shannon M Wallet, Jean-François Roulet, Mario Alexandre Coelho Sinhoreti, Dayane Oliveira

**Affiliations:** 1 University of Florida College of Dentistry Department of Restorative Dental Science Gainesville FL USA University of Florida, College of Dentistry, Department of Restorative Dental Science, Gainesville, FL, USA.; 2 University of Florida College of Dentistry Department of Oral Biology Gainesville FL USA University of Florida, College of Dentistry, Department of Oral Biology, Gainesville, FL, USA.; 3 Universidade Estadual de Campinas Faculdade de Odontologia de Piracicaba Departamento de Odontologia Restauradora Piracicaba SP Brasil Universidade Estadual de Campinas (UNICAMP), Faculdade de Odontologia de Piracicaba, Departamento de Odontologia Restauradora, Piracicaba, SP, Brasil.

**Keywords:** Curing lights, Light curing unit, Light curing, Polymerization, Temperature

## Abstract

**Objectives:**

To evaluate the effect of wavelength and radiant emittance on light transmittance and heat generation through dental resin-based composites.

**Methodology:**

Light-emitting diodes on the blue, green, and red wavelength spectra were assembled and characterized using a spectrometer (MARC Resin Calibrator, BlueLight Analytics). Voltage (V) and amperage (A) from each LED was set up to emit a 500-, 1000-, 2000-, 3000-, or 4000-mW/cm^2^ radiant emittance. A self-cured resin-based composite model was fabricated in two pastes, one with benzoyl peroxide and another with ethyl 4-dimethylaminobenzoate. The two pastes were mixed and placed into a mold (ø=10 mm, 2 mm thick) until completely hardened. A power analysis was conducted to determine the sample size to provide a power of at least 0.8 and α=0.05. Light transmittance through the 2-mm thick composite-based sample was evaluated using a spectrometer (n=10). Heat generation (^o^C) in the resin-based composite sample induced by the exposure to the tested wavelength spectra and radiant emittances were recorded using an infrared camera (FLIR ONE PRO, FLIR Systems) (n=10). Statistical analyses were performed using analysis of variance and the Tukey's test for multiple comparisons.

**Results:**

Light transmission systematically increased based on radiant and wavelength emittances (p<0.0001). Heat generation was directly proportional to radiant emittance but indirectly proportional to wavelength emittance (p<.0.0001).

**Conclusions:**

Despite its limitations, this study found that increasing wavelength emittance seems to configure a great alternative to increase light transmittance through resin-based composite restorations while reducing heat generation.

## Graphical abstract

**Figure:**
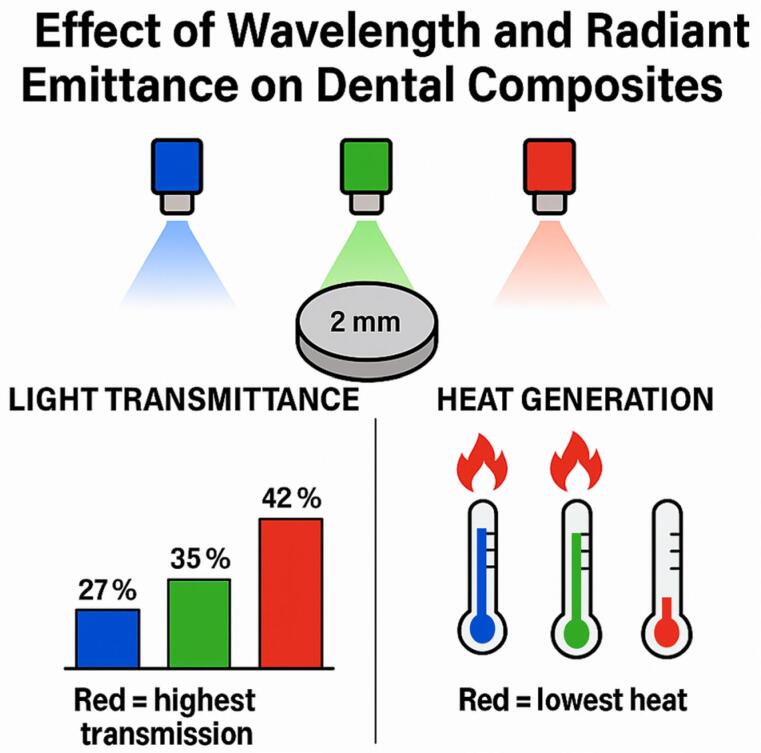

## Introduction

Light emitting diodes are used in dentistry for light curing resin-based composite restorations.^1,2^ The adequate light curing of these restorations must be performed to attain proper mechanical properties and clinical longevity.^1^ Resin-based composite restorations are likely to be deemed as failing prematurely when depth of cure fails to occur throughout the thickness of the entire material due to marginal discrepancies in the surface margins of the restoration.^3,4^ Depth-of-cure issues can be related to many causes, including malposition, quality, or malfunctioning of the light curing unit and its own profile characteristics,^5^ which can lead to the resin-based composite material receiving varying radiant exposures across the restoration.^1^ This results in uneven polymerization throughout the resin-based composite, especially in its deeper parts.^6,7^

The radiant emittance received from the light curing unit and exposure time constitute two of several factors that can influence the degree of conversion of a resin-based composite.^8–10^ Moreover, the depth of cure will also depend on the fact that wavelengths have differing penetration capabilities.^6,7^ To enable a higher degree of conversion, many dentists increase the radiant emittance output of their light curing unit and/or exposure length.^7,11^ Raising the radiant emittance output of a light curing unit increases the depth of the cure for the resin-based composite due to a higher light transmittance through the material.^12^ With higher light transmittance through the material, monomers and photoinitiators in the deeper parts of the resin-based composite can react and continue the polymerization process in depth.^7^ On the other side, a higher radiant emittance output and longer exposure can harm pulpal and gingival tissues^13–15^ since any unabsorbed energy delivered to the material is dissipated as heat to surrounding tissues.^13^

LED curing units started as what are now called monowave units with a single peak spectral emission within the visible blue light range. However, these monowave light curing units could only activate photoinitiators within this absorption spectra, such the camphorquinone.^16^ Camphorquinone is a commonly used photoinitiator in dentistry. It has a 360-510-nm absorption spectra range and a maximum absorption peak at 468nm.^17^ Newer LED curing units—called multiwave—were introduced. Their LED chips emit blue light and also emit wavelengths within the visible violet light range to activate other photoinitiators, such as ^2,4,6^—trimethylbenzoyl-diphenylphosphine oxide (TPO) and bisacylphosphine oxide (BAPO).^16^ TPO has a 380-425-nm absorption spectra range AND a maximum absorbance peak at about 400nm (or 379-381nm), whereas BAPO has a 365-416-nm absorbance spectra range and a maximum absorbance peak at about 400 nm (or 371-398nm).^17^ Thus, most multiwave LED light curing units emit at least two peaks in distinct wavelengths, having a much broader spectral emittance range.^16^ Since shorter wavelengths are inherently more energetic, violet light is expected to carry more energy than blue light when delivered at similar radiant emittances.^7,12,18^ This can increase heat generation. Thus, when used inadvertently, multiwave light-curing units may cause more harm than monowave-curing ones.^12,16^

Additionally, the shorter the wavelength, the lower its light transmittance capability, which affects the depth of cure of light-cured resin-based composites when activated with multiwave light curing units.^7,18^ The blue light spectra range from 420 to 495 nm, whereas the violet light ones, from 380 to 420 nm.^19^ The inherent lower light transmittance of violet light in relation to blue light can cause differences in the degree of conversion throughout the resin-based composite and affect the depth of cure of the restoration.^7,18^

In contrast, longer wavelengths show opposing characteristics, offering greater light transmittance capabilities that can benefit adequate depth of cure in light-cured resin-based composites.^7,18^ Moreover, these wavelengths are less energetically charged than violet and blue light. Thus, if energetically balanced, longer wavelengths could mitigate potential harm to the pulp and gingival tissues by generating less heat.^20^ Therefore, this study aimed to evaluate the effect of wavelength and radiant emittance on light transmittance and heat generation through dental resin-based composites. The tested hypotheses in this study were that (1) LED spectral and radiant emittance would influence light transmittance through the dental resin-based composite and that (2) LED spectral and radiant emittance would influence heat generation through the dental resin-based composite.

## Methodology

### LED characterization

LEDs emitting blue, green, and red wavelength spectra (Chanzon, Shenzhen, China) were coupled to a power supply (Tekpower, Montclair, CA, USA) that could control their voltage (V) and amperage (A). Voltage and amperage were set up to each LED to emit a 500-, 1000-, 2000-, 3000-, or 4000-mW/cm^2^ radiant emittance. LED output stability and the mean radiant emittance (mW/cm^2^) according to the wavelength ranges (nm) from each LED was characterized using a spectrometer (MARC Resin Calibrator, BlueLight Analytics, Halifax, Nova Scotia, Canada).

### Model resin-based composite formulation

A single 2-mm thick resin-based composite sample with a 10-mm diameter was used for all analyses. A self-cured resin-based composite model was made for standardization. First, a monomer blend containing 10 wt% of bisphenol A diglycidylmethacrylate, BisGMA (Sigma Aldrich, St. Louis, MO, USA), 10.5 wt% of ethoxylate bisphenol A diglycidylmethacrylate, BisEMA (Sigma Aldrich, St. Louis, MO, USA), 10.5 wt% of urethane dimethacrylate, UDMA (Sigma Aldrich, St. Louis, MO, USA), and 2 wt% of triethylene glycol dimethacrylate TEGDMA (Sigma Aldrich, St. Louis, MO, USA) was mixed for 1 minute at 3000 rpm using a centrifugal mixer (SpeedMixer, DAC 150.1 FVZ- K, Hauschild Engineering, Hamm, North Rhine-Westphalia, Germany). To this resin blend, two pastes were made, one containing 2 wt% of benzoyl peroxide (Sigma Aldrich, St. Louis, MO, USA) or 2 wt% ethyl 4-dimethylaminobenzoate, EDMAB (Sigma Aldrich, St. Louis, MO, USA). Subsequently, 13 wt% of 50 nm fumed silica (Nippon Aerosil Co. Ltd., Tokyo, Japan) and 52 wt% of 0.7 μm barium borosilicate glass filler (Esstech Inc., Essington, PA, USA) were added to each paste, first by pre-mixing the fumed silica filler with the monomer blend for 30 seconds at 3000 rpm, followed by the barium borosilicate glass filler for 1 minute at 3500 rpm. Then, the resin composite pastes were then mixed one final time for 1 minute at 3500 rpm under vacuum to eliminate porosities. The model resin composite pastes containing benzoyl peroxide or EBMAB were mixed, placed in a mold (ø=10 mm, 2 mm thick), and kept for 48 hours until completely cured. By using the same model restorative material formulation and sample across all tested wavelength ranges and radiant emittances we were able to isolate the specific effects of wavelength and radiant emittance on light transmittance. Moreover, the use of a self-cured, fully polymerized composite prior to testing aimed to eliminate thermal effects resulting from the polymerization reaction and to avoid refractive index changes that could alter light transmittance, thereby isolating the effects of wavelength and radiant emittance.

### Light transmittance analysis

The overall radiant emittance (mW/cm^2^) of the blue, green, or red spectra transmitted through the resin-based composite sample was evaluated using a spectrometer (Marc Resin Calibrator, BlueLight Analytics, Nova Scotia, Canada). The resin-based composite sample was interposed between the LED being tested and the input sensor of the spectrometer (⌀=3.9 mm). For each spectrum, the following radiant emittances were tested: 500, 1000, 2000, 3000, or 4000 mW/cm^2^ (n=10). A custom software (BlueLight Analytics, Nova Scotia, Canada) was used to transfer all databases to Excel files (Microsoft Corp., Redmond, WA, USA). Withal, the results were exported to a graphing software (OriginPro, OrigenLab Co., Northampton, MA, USA) to create separate image files to illustrate the light transmittance of the different spectra through the resin-based composite sample according to the tested radiant emittance.

### Thermal analysis

The overall heat generation (^o^ C) in the resin-based sample induced by the light exposure to the tested wavelength spectra and radiant emittances (n=10) were recorded live using an infrared camera (FLIR ONE PRO, FLIR Systems, Wilsonville, OR, USA) with a<0.10 ^o^C thermal sensitivity and 150 mK. Initial temperature (37 ^o^C) and humidity (75%) parameters were standardized to simulate clinical conditions using a humidity and temperature meter (Extech 445580, Extech, Waltham, MA, USA). Then, a software suite (FLIR Tools, FLIR SYSTEMS, Willsonville, OR, USA) was used to transfer the video database to a data-sheet in Excel files (Microsoft Corp., Redmond, WA, USA) and to collect image files to illustrate temperature changes.

### Statistical analysis

A power analysis was conducted to determine sample size and power of at least 0.8 at a significance level of α=0.05. Randomization was statistically analyzed by the Shapiro-Wilk normality test, analysis of variance, Brown-Forsythe, and Bartlet's variance analysis tests. Data normality and homoscedasticity were evaluated by the Shapiro-Wilk and Lavine's tests, respectively. Statistical analyses were performed by analysis of variance and the Tukey's test for multiple comparisons.

## Results

### Light curing unit characterization

**Figure 1:**
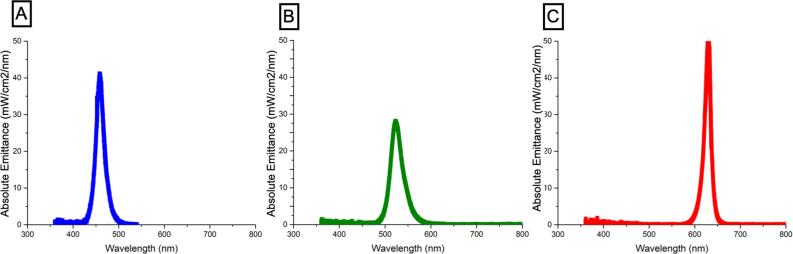
Spectral emittance (mW/cm^2^/nm) for each LED emitting several spectra: blue (A), green (B), and red (C) against wavelengths (nm).

Table 1 describes the voltage (V) and amperage (A) set up for each LED to emit the 500-, 1000-, 2000-, 3000-, or 4000-mW/cm^2^ radiant emittances. Figure 1 illustrates the spectral emittance (mW/cm^2^/nm) for each LED emitting the blue (A), green (B), and red (C) spectra, plotted against wavelength (nm). The LED emitting the blue spectra showed a single peak emission near 470nm; that emitting the green spectra, a single peak emission near 540nm; and that emitting the red spectra, a single peak emission near 620nm.

**Table 1:** Voltage (V) and amperage (A) set up for each LED to emit a 500-, 1000-, 2000-, 3000-, or 4000-mw/cm^2^ radiant emittance.

LED Spectra	Radiant Emittance (mW/cm^2^)	Voltage (V)	Amperage (A)
Blue	500	7,990	0,070
	1000	8,290	0,145
	2000	8,800	0,380
	3000	9,110	0,500
	4000	9,480	0,749
Green	500	7,250	0,127
	1000	8,210	0,285
	2000	9,030	0,673
	3000	9,480	1,223
	4000	9,850	1,923
Red	500	5,700	0,213
	1000	5,940	0,423
	2000	6,150	0,890
	3000	6,590	1,473
	4000	7,180	2,363

When using the setups in Table 1, the LED emitting the blue spectra showed 499 (±.2), 998 (±2.1), 2013 (±1.6), 3016 (±14.1), and 4033 (±37.7) mean radiant emittances (mW/cm^2^); that emitting the green spectra, 506 (±2.5), 1005 (±0.4), 2002 (±5.3), 3005 (±4.2), and 4024 (±37.8); and that emitting the red spectra, 499 (±1.5), 1000 (±0.4), 2007 (±8.7), 3019 (±15.0), and 4006 (±40.7).

### Light transmittance analysis

**Figure 2:**
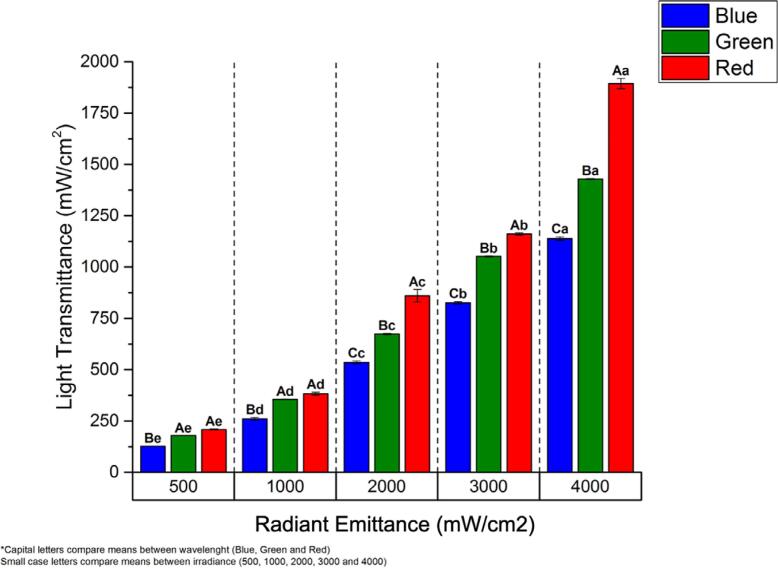
Overall radiant emittance (mW/cm^2^) of the blue, green, and red spectra transmitted through the resin-based composite sample according to radiant emittance setups (500, 1000, 2000, 3000, or 4000 mW/cm^2^). Capital letters compare the means between wavelengths. Lowercase letters compare means between irradiance setups.

Figure 2 illustrates the overall radiant emittance (mW/cm^2^) of the blue, green, and red spectra transmitted through the resin-based composite sample according to the radiant emittance setups (500, 1000, 2000, 3000, or 4000 mW/cm^2^). As can be observed, regardless of the emitted spectra, the overall light transmission systematically increased based on radiant emittance (*p*<0.0001). Regardless of radiant emittance, overall light transmission also systematically increased with wavelength (*p*<0.001). For the blue spectral transmission (short wavelength), about 26.6% of the radiant emittance was transmitted, whereas for the green one (medium wavelength), about 34.78% and for red one (long wavelength), about 41.72%.

### Thermal analysis

**Figure 3:**
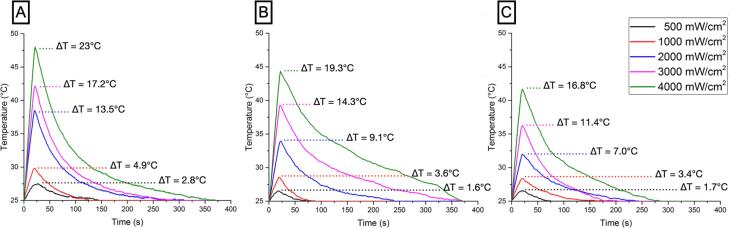
Heat fluctuation (^o^C) in the resin-based composite sample caused by exposure to blue (A), green (B), and red (C) spectra and radiant emittance setups (500, 1000, 2000, 3000, or 4000 mW/cm^2^).

Figure 3 illustrates the overall heat generation (^o^ C) in the resin-based composite sample caused by exposure to the blue, green, and red spectra and radiant emittance setups (500, 1000, 2000, 3000, or 4000 mW/cm^2^). As observed in Figure 3, regardless of the emitted spectra, overall heat generation was directly proportional to radiant emittance. The higher the radiant emittance, the higher the overall heat generation in the resin-based composite sample. However, regardless of radiant emittance, overall temperature changes were indirectly proportional to wavelength emittance (*p*<0.001).

**Figure 4:**
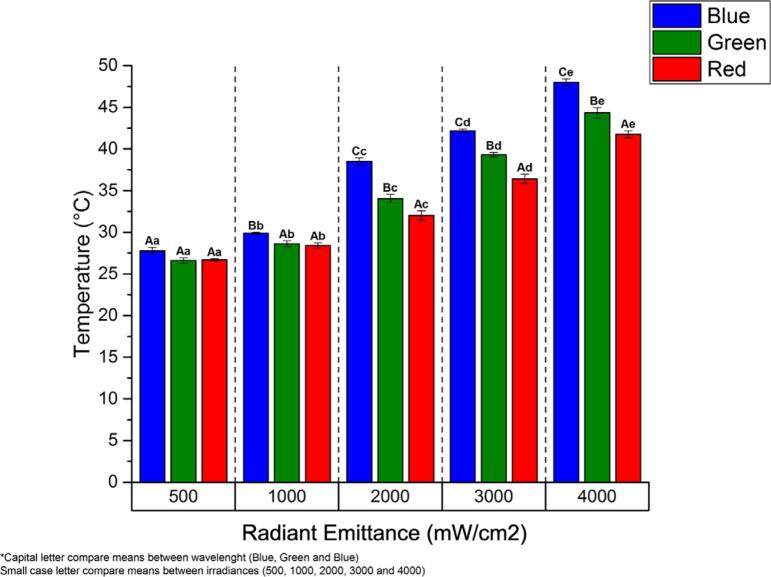
Overall heat generation (C) in the resin-based composite sample caused by exposure to blue, green, and red spectra and radiant emittance setups (500, 1000, 2000, 3000, or 4000 mW/cm^2^). Capital letters compare the means between wavelengths. Lowercase letters compare means between irradiance setups.

**Figure 5:**
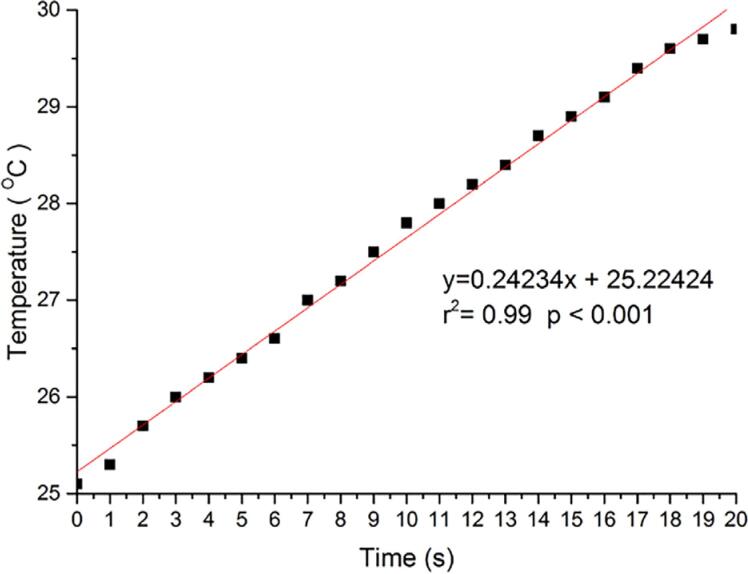
Linear regression analysis of temperature (°C) over time (seconds) during light exposure using the blue spectrum (1000 mW/cm^2^). This condition was chosen as a representative example to show the linear relationship between exposure time and thermal increase. Data correspond to the condition in Table 2.

Figure 4 shows that regressing the results from heat generation (^o^C) against the time of exposure (s) can predict the time for the temperature to increase more than 5.5 ^o^C. Figure 5 shows the relationship between temperature (°C) and time (seconds) for the blue light spectrum at 1000 mW/cm^2^, whereas Table 2 describes the exposure (s) necessary to increase the temperature by more than 5.5 ^o^C when under the blue, green, and red spectra and the radiant emittance setups (500, 1000, 2000, 3000, or 4000 mW/cm^2^).

**Table 2:** Time of exposure at temperature increases up to more than 5.5^o^C.

Radiant Emittance (mW/cm^2^)	LED spectra		
	Blue	Green	Red
500	53 sec	61 sec	72 sec
1000	22 sec	28 sec	31 sec
2000	7 sec	13 sec	15 sec
3000	6 sec	8 sec	9 sec
4000	5 sec	6 sec	6 sec

## Discussion

As observed in the results, LED spectral emittance directly influenced light transmittance and temperature change in the dental resin-based composite. Thus, the first research hypothesis that LED spectral and radiant emittance would influence light transmittance through the dental resin-based composite was accepted. Our results showed that light transmittance increased with radiant emittance regardless of light wavelength since a higher radiant emittance indicates a higher energy from the curing light, increasing spectral emission throughout the resin-based composite.^7^ Additionally, light transmittance increased with the wavelength spectra. Specifically, only ± 6.6% of the radiant emittance from the blue spectra was transmitted (compared to ±34.78% from the green spectra and ±41.72% from the red spectra). This shows that increasing the wavelength of spectral emission can enhance light transmittance, in which red spectra improved transmittance by up to 15% in relation to blue spectra and by 8% in relation to green spectra.

In resin-based materials, the degree of conversion is related to the amount of light absorbed by photoinitiators.^21^ Upon absorbing light, the photoinitiators generate radicals that initiate the polymerization reaction.^21^ Violet light, a shorter wavelength than blue light, shows lower penetration and poorer in-depth transmission through composite materials than blue light.^7^ Studies have shown that violet light has a very low transmission through composite materials, which reduces its depth of cure by up to 1 mm.^7,22,23^ The lower penetration of shorter wavelengths is due to a phenomenon called Rayleigh scattering, which occurs when light interacts with particles smaller than the wavelength of the light. The shorter the wavelength, the closer the wavelength is in size to these particles, increasing scattering when compared to longer wavelengths.^18^ As scattering increases, the more the light is attenuated in depth.^7^ With light attenuation, the energy reaching the deeper parts of the composite is insufficient for the photoinitiators to continue the polymerization.^21^ This has several clinical implications, including the restoration of Class II proximal boxes, for example.^1^ The proximal box areas of Class II preparations average around 4–5 mm deep, which can challenge restorations with resin-based composites containing alternative photoinitiators.

Another clinical implication is that the light-curing unit spectral emittance distribution (the beam profile) and its impact on polymerization homogeneity must be considered.^24^ Light-curing units often vary in radiant and spectral emittances between the center and the edges of the light tip.^24^ However, these variations are much more accentuated in multiwave curing lights due to the several LED chip arrangements within the curing tip. Thus, the position of the curing light relative to the resin-based composite can affect polymerization as different parts of the composite receive varying levels of spectral and radiant emittances.^24^ In clinical practice, this is less concerning when using composites only containing camphorquinone. However, for materials containing alternative photoinitiators, such as TPO or BAPO, these beam profile discrepancies become much more problematic. To mitigate this risk, clinicians should position the curing light as centrally as possible over each increment being cured, ensuring that the most uniform and spectrally balanced region of the beam (typically the center) is aligned with the area of interest. This practice is particularly critical when using multiwavelength curing lights in deep Class II restorations to avoid undercuring at the margins or base of the proximal box.

Shorter wavelengths, such as violet and blue lights, are more energetic and carry more photon energy than red or green light when delivered at similar radiant emittances.^7,12,18^ Initially, this was thought to be advantageous as higher energy would start the reaction quicker and enhance the degree of conversion.^25^ However, any photon energy the photoinitiators in the composite failed to absorb is released as heat into the surrounding tissues, potentially causing adverse effects on the pulpal and gingival tissues.^12,15^ In contrast, longer wavelengths have lower photon energy, resulting in little to no excess of photon energy being dissipated as heat to surrounding tissues.^12^ Although violet and ultraviolet wavelengths are relevant for activating alternative photoinitiators such as TPO and BAPO, their use has been extensively studied. Findings show that they result in lower light transmittance and higher heat generation due to their shorter wavelengths.^7,12,18^ Therefore, this study focused on longer wavelengths (green and red) to explore their potential advantages over the commonly used blue light regarding light transmittance and thermal safety.

The second hypothesis that LED spectral and radiant emittance would influence heat generation through the dental resin-based composite was also accepted. As observed in the results, at a 1000-mW/cm^2^ radiant emittance, blue light increased the local temperature by more than 5.5 ^°^C in 22 seconds, whereas green light did so in 28 seconds and red light in 31 seconds. At 2000 mW/cm^2^, blue light reached the same temperature increase in only seven seconds; green light, in 13 seconds; and red light, in 15 seconds. These findings are in line with the principle that higher radiant emittances deliver more photon energy, which, if unabsorbed, is dissipated as heat, increasing temperature.^7,12,26^ Rises in temperature can also be partially attributed to the exothermic reaction during polymerization.^1^ These results are significant as it is important to consider that resin composites are typically cured for 20 seconds, and the radiant emittance of modern high-power light curing units often exceeds 1000 mW/cm^2^.^1^ Moreover, resin composites are usually cured in multiple increments, resulting in several consecutive 20-second exposures.^1^

Increases in temperature harm soft tissues, compromising their structural integrity.^12^ The heat generated by light exposure can damage cells by disrupting their metabolic processes and protein stability, impairing cellular function and reducing viability. Thermal stress can denature proteins, alter enzyme activity, and compromise membrane integrity, further contributing to cellular damage.^15,27^ In addition to thermal effects, blue light exposure reduces total protein production and alkaline phosphatase activity in cells.^27^ Almeida, et al.^27^ (2017) have shown that blue light is absorbed by porphyrins in hemoglobin and myoglobin, leading to the metabolic breakdown of these products and the formation of toxic byproducts. This suggests that blue light toxicity extends beyond heat generation, further amplifying its detrimental effects on cellular health.^27^

In contrast, studies have shown that red light enhances the metabolism of cells and promotes the synthesis of collagen-rich matrix and proteins, which are essential for tissue repair and regeneration.^27,28^ Additionally, red light stimulation has been associated with the formation of mineralized tissue, leading to reduced dentinal sensitivity and the natural accumulation of tertiary dentin around the pulpal tissue.^28^ Thus, these cellular responses can play an essential role in supporting the repair of the pulp-dentin complex following damage caused by caries.^28^ However, note that photobiomodulation is dose-dependent. While red light has shown favorable cellular effects, excessive radiant exposure (even at beneficial wavelengths) may surpass the therapeutic window and result in unintended cellular stress or inhibition. Therefore, careful control of radiant exposure is essential when clinically applying these findings to ensure that the benefits of photobiomodulation fail to be offset by overexposure.

A limitation of this study is that all results were obtained in a simulated environment. As such, it is uncertain whether these findings would fully translate to real-world conditions within the oral cavity. Particularly, the material in this study was standardized to ensure consistency in specimen design and measurement reproducibility. However, resin formulations may slightly vary in optical and thermal behavior, which could influence the results. Moreover, although the composite used in this study was 10 mm in diameter, which exceeds the typical size of clinical restorations, this dimension was selected to ensure full coverage by the curing light tip and consistent light exposure across all specimens. However, such larger surface area may have facilitated greater lateral heat dissipation, potentially underestimating possible peak temperatures in confined clinical cavities. Lastly, the curing light in this study was positioned in direct contact with the resin surface. Clinically, however, light curing is often performed at a distance of 2 mm or more due to limited access. This increased distance reduced the irradiance reaching the material and decreased heat generation. Note that, in clinical conditions, dentin serves as a natural thermal barrier, helping to mitigate temperature rises from light-curing procedures. Thus, future *in vivo* studies are needed to validate the applicability of these results.

Despite these limitations, the findings in this study suggest that red light seems to be a safer alternative to blue and violet light in dental applications, offering potential benefits with reduced risks to soft tissues. These results underscore the importance of selecting curing lights and protocols that optimize depth of cure while minimizing adverse thermal effects, guiding current clinical practices and the development of the next-generation of curing devices.

## Conclusions

Despite its limitations, this study found that increasing the wavelength emittance of curing lights seems to offer a great alternative to increase the light transmittance through resin-based composites and reduce heat generation.

## Data Availability

All data generated or analyzed in this study are included in this published article.
